# Reduced Neural Synchrony in Patients with Restless Legs Syndrome during a Visual Oddball Task

**DOI:** 10.1371/journal.pone.0042312

**Published:** 2012-07-27

**Authors:** Jeong Woo Choi, Deokwon Ko, Gwan-Taek Lee, Ki-Young Jung, Kyung Hwan Kim

**Affiliations:** 1 Department of Biomedical Engineering, College of Health Science, Yonsei University, Wonju, South Korea; 2 Department of Neurology, Korea University Medical Center Anam Hospital, Korea University College of Medicine, Seoul, South Korea; University of Maryland, United States of America

## Abstract

**Background:**

Restless legs syndrome (RLS) is a sensorimotor neurological disorder characterized by an irresistible urge to move the legs. It has been reported that RLS patients show cognitive deficits, presumably due to hyperactivity causing loss of attention, or malfunctions in the frontal region resulting from sleep deprivation. However, the mechanism underlying cognitive deficits in RLS patients is mostly unknown. As an effort to clarifying this, we investigated the differences in neural activity and phase synchrony between healthy controls and RLS patients during cognitive task performances.

**Methodology/Principal Findings:**

Seventeen female drug-naive RLS patients were enrolled in the study, and an age-matched group of thirteen healthy female volunteers served as controls. Multichannel event-related potentials (ERPs) were recorded from RLS patients and normal controls while performing a visual oddball task. In addition to conventional analyses of ERP waveforms and spectra, interregional gamma-band phase synchrony (GBPS) was investigated to observe the differences in interregional neural synchronies between normal and RLS patient groups. Strong GBPS was observed primarily between anterior and posterior regions along the midline for both groups. Along with significant reduction and delay of P300 ERP and induced gamma-band activity (GBA), the GBPS was considerably decreased in RLS patients compared to normal subjects, especially at frontal region.

**Conclusions:**

Overall, our results support that cognitive dysfunction in RLS patients is associated with reduced interregional neural synchrony as well as alterations in local neural activity.

## Introduction

Restless legs syndrome (RLS) is a sensorimotor neurological disorder characterized by an irresistible urge to move the legs. Despite symptoms in the legs, a large amount of evidence indicates that the RLS is a disorder of the central nervous system [1]–[3]. In support of this, it has been recently shown that cognitive deficits are associated with RLS [4], [5], which have been attributed to chronic partial sleep deprivation affecting prefrontal brain functions [4], or the inattentiveness caused by RLS symptoms [6]. Contradictory results can be found in the literature [5], and thus, it is likely that neither sleep loss nor inattentiveness alone can explain the cognitive deficits observed in RLS patients.

A few studies on brain function using electrophysiological or neuroimaging methods were performed to identify the mechanism of cognitive dysfunction in RLS. Functional or anatomical abnormalities in sensorimotor cortical areas in RLS were demonstrated [7]–[9]. Schober et al. [7] showed that post-movement beta oscillations in the motor cortex were significantly elevated in RLS patients. Tyvaert et al. [8] observed that the amplitudes and durations of event-related synchronization/desynchronization in the beta and mu bands were greater during the symptomatic period for RLS patients compared to controls. Furthermore, significant neuroanatomical alteration in RLS patients was reported, including regional decreases in gray matter volume in the primary somatosensory and motor cortices [9].

Recently, we reported a result of event-related potential (ERP) study for the purpose of the identification of the mechanism underlying cognitive deficits in RLS [6]. The P300 ERP component was significantly reduced and delayed in RLS patients during a visual oddball task, and its latency was correlated with a bothersomeness score. We also observed that beta-band power was increased considerably compared to normal controls, primarily in the prefrontal region. Overall, our recent results provide an electrophysiological evidence for cognitive deficits in RLS patients, supporting previous behavioral studies regarding RLS-associated prefrontal dysfunction.

In addition to local neural activities revealed by ERP analysis, exploring neural synchrony within and between task-relevant cortical regions may provide additional valuable insights into the mechanisms of neural information processing. This can be accomplished by analyzing event-related spectral perturbation (ERSP) and interregional phase synchronization between electroencephalograms (EEGs) from multiple cortical regions [10]–[12]. Simultaneous observation of both large- and small-scale neural associations is achievable, which may lead to a more complete view of cortical information processing. Based on the hypothesis that the cognitive deficits observed in RLS patients are partially due to local and global synchrony in neural activity, we investigated differences in gamma-band activity (GBA) and interregional gamma-band phase synchrony (GBPS) between normal subjects and RLS patients. A considerable reduction in GBPS as well as GBA was observed in RLS patients during a visual oddball task. Furthermore, graph theoretical analysis of GBPS pattern revealed a significant change in functional cortical networks associated with RLS. A subset of results on the ERP analysis has been published recently [6].

## Methods

### Subjects

Seventeen female drug-naive RLS patients (53.7±9.6 years) were enrolled in the study. All patients participated in a standardized interview, including a structured sleep questionnaire and clinical neurological examinations by a neurologist experienced in sleep medicine and RLS (KJ). The sleep questionnaire contains the Global Sleep Assessment Questionnaire [13], the Pittsburgh Sleep Quality Index [14], the Epworth Sleepiness Scale [15], and the Insomnia Severity Index [16]. It includes questions on sleep habits and medication history as well. RLS was diagnosed following the criteria proposed by the International RLS Study Group [17]. Subjects responding affirmatively to all 4 questions were considered to have RLS.

Patients were excluded if they had RLS mimics (e.g. cramps, neuropathies, arthritis, and positional discomfort [18], [19]) or secondary cause of RLS including a history of taking drugs known to cause RLS (e.g., neuroleptics, antidepressants, or antihistamines), a relevant neurological or psychiatric disorder, or a history of sleep-related disorders other than RLS-related insomnia. Hemoglobin, blood glucose and serum levels of creatinine, iron/ferritin, and thyroid hormones were checked in all patients. RLS severity was determined using the International RLS Severity Scale (IRLS) [20].

An age-matched group of 13 healthy female volunteers without any sleep disturbance served as controls (54.6±7.6 years). Participants were recruited on a voluntary basis by local advertisement. All the subjects completed the same sleep questionnaire including the Global Sleep Assessment Questionnaire, the Pittsburgh Sleep Quality Index, the Epworth Sleepiness Scale, and the Insomnia Severity Index and were questioned the four essential RLS diagnostic criteria of the International Restless Legs Syndrome Study Group (IRLSSG). Subjects were included as healthy control in the present study only if they were normal range in a sleep questionnaire and not RLS in the IRLSSG diagnostic criteria.

All subjects signed written informed consents. The experimental protocol was approved by the Institutional Review Board of Korea University Medical Center.

### Experiments

In accordance with the international 10–20 system, electroencephalograms (EEGs) were recorded using 27 electrodes with extended coverage of lower temporal region (F9/10, T9/10, and P9/10). The reference electrode was set to linked-mastoid electrodes. Two electrooculogram (EOG) channels (placed on the left and right outer canthi) were added to confirm eyeball movements to remove ocular artifacts. Impedances of all electrodes were maintained below 5 kΩ. A bandpass filter (0.1–100 Hz) and 60 Hz notch filter were applied.

All experiments were performed at 10 am. Spontaneous EEGs were recorded under waking-rest conditions for about 5 min prior to ERP sessions, and repeated 6 times with eyes alternately closed (for 20 s) and open (for 20 s) to ascertain alertness. In addition, event-related potentials (ERPs) were recorded during the visual oddball paradigm. A commercial software program (PRESENTATION; Neurobehavioral systems, Berkeley, CA) was used to present stimuli to patients on a 17-inch LCD monitor. Stimuli consisted of a white equilateral triangle (50 mm, standard stimulus) and a white square (50 mm, target stimulus) on a black background. The distance between subjects' eyes and the monitor was approximately 75 cm, and the visual angle was 1.91°. The stimuli were presented for 200 ms in a randomized order at a standard to target ratio of 4∶1. Interstimulus intervals were fixed at 1200 ms. Black screens were presented otherwise. Subjects were asked to respond only to target stimuli by pressing a button as quickly as possible. The entire experiment for each subject was divided into two blocks with 400 stimuli presented during each block. Subjects rested for 5 min between blocks.

Experimental sessions generally took 25–30 min per subject. The Stanford sleepiness scale (SSS) [21] and the visual analog scale (VAS) for bothersomeness [22] (from 0 to 10 points) were used to assess either RLS symptoms or experimental procedures immediately after ERP sessions. In addition, the SSS was also measured just before sessions.

### Preprocessing and ERP Analysis

We removed single-trial waveforms from further analysis if visual inspection confirmed that they were severely contaminated from non-stereotyped artifacts such as drift or high-frequency noise. In addition, EEG segments were excluded from further analysis if the absolute value of the EOG exceeded 100 µV. The average number of epochs recorded per patient for standard and target stimuli were 485.2±98.7 and 123.7±22.8, respectively.

After the preprocessing, averaged ERPs were obtained during the −200∼800 ms interval after the stimulus onset. Baselines were corrected by subtracting the root mean square of the signal during prestimulus interval. Several ERP components were identified according to polarity, latency, and topography from grand-averaged ERP waveforms, and then analyzed in detail as reported in Jung et al [6]. ERP latencies and amplitudes were measured relative to prestimulus baselines. The P300 component was defined as a positive peak between 325 and 450 ms poststimulus.

Statistical comparisons were performed by repeated measures analysis of variance (ANOVA). Within-subject variables were stimulus type (two levels: standard and target) and location (three levels: frontal, central, and parietal), and the between-subject variable was the group (i.e., RLS vs. control). The Greenhouse-Geisser correction for multiple comparisons was employed when appropriate and corrected *p* values were reported. The variables showing significant main effects were further analyzed with *post hoc t*-test with Bonferroni correction.

### Event-related spectral perturbation (ERSP)

To examine the temporal evolution of spectral characteristics by ERSP, a continuous wavelet transform (CWT) with complex Morlet wavelet was applied to single trial and averaged ERPs [23] for the target stimuli. Evoked (or phase-locked) gamma-band activity (GBA) was obtained from the ERSP of averaged ERPs aligned to the stimulus onset. Induced GBA was obtained by averaging ERSP patterns of each single trial, as illustrated in Hermann et al. [24]. The time-frequency windows of interest were determined by visual inspection of the ERSP maps so that the power was notably increased within those windows. The mean amplitude and peak latency of the GBA within those windows were statistically compared between the locations and groups using two-way ANOVA with location as a within-subject variable (three levels: frontal, central, and parietal), and group as a between-subject variable (RLS and control).

Possible association between the local neural synchrony and clinical measures was investigated by Pearson's correlation coefficient between the amplitude/latency of GBA and clinical measures within each group.

### Phase synchronization in gamma-band

In order to analyze interregional GBPS, the phase synchronization index was calculated [11]. Single-trial ERP signals were first transformed into narrowband signals in the gamma-band by bandpass filtering (30–50 Hz). The instantaneous phase 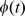 for each time point *t* was calculated from the narrowband signal *x*(*t*) and its Hilbert transform 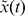 as follows [10], [11]:


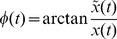


To quantify the degree of phase synchronization, the phase-locking value (PLV) between two electrodes *j* and *k* was calculated for each time point *t* by averaging the phase difference over *N* trials as follows [11]:


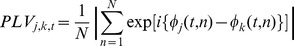


Here *N* represents the total number of trials and *n* denotes a specific trial within these; 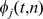 designates the phase of signals of electrode *j* at time *t* of the *n^th^* trial.

We then determined whether the PLVs between two specific electrodes indicated significant phase synchronization. For this purpose, we devised a double-threshold strategy based on two criteria [25]. The first step involved checking whether the PLV change under investigation was meaningful with respect to the PLVs of surrogate data, which were obtained from random shuffling of trials [11]. Surrogate data were obtained from 200 random shufflings of trials for each electrode pair. Using the distribution of PLVs calculated from the surrogate data, we were able to determine the level of significance, as explained by Lachaux et al. [11] and Doesburg et al. [26]. We set the significance level at 1%; i.e., we decided that the phase synchronization was significantly increased if it was higher than the first percentile of the PLV values from surrogate data.

The second criterion was to determine whether the PLV increased significantly during task execution compared to the prestimulus baseline PLV. After obtaining the baseline distribution of PLVs during the prestimulus period (200 samples) by averaging across trials, we set the level of significance as that of the first step to 1%. In other words, we determined whether the phase synchronization significantly increased such that it became higher than the first percentile of the PLV values obtained from the EEG during the prestimulus period.

### Graph theoretical analysis of phase synchrony map

The pattern of interregional phase synchronization can be visualized as graphs consisting of nodes and edges. Thus, it can be quantitatively analyzed using graph theory [27]. The nodes of a graph are determined by the electrodes included in phase synchronization analysis, and the edges are determined by the electrode pairs showing significant phase synchronies. The graph is quantitatively characterized by a symmetric adjacency matrix. The undirected binary element *a_ij_* of the adjacency matrix represents a connection between two nodes *i* and *j*, and it is designated 1 or 0 according to the presence or absence of a significant connection between the two nodes.

A set of numerical measures for the property of graphs can be derived from the adjacency matrix [27]. Clustering coefficient *C* is used as an index of local connectivity. It is defined by the following equation:


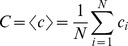


where *c_i_* is the ratio of the number of existing edges between neighbors, which are connected to electrode *i* by an edge, and the maximum possible number of edges between its neighbors. The clustering coefficient *C* can be interpreted as a measure of the network's resilience to error, since in case of high clustering, even if a node is lost, its neighbors remain connected.

In addition to the measure of local connectedness, characteristic path length *L* provides an important measure characterizing the global connectedness. It is defined as follows:


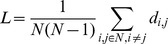


where the path length, *d_i, j_* is the minimum number of edges that must be traversed to go from node *i* to *j*. Because *L* is the average path length between all possible pairs of *N* nodes, it represents global connectivity that describes how well integrated a graph is, and how easy and fast it is to transport information in the network [28].

The *C* and *L* are dependent on the number of edges of each graph, and thus they may be significantly affected by the number of connections, rather than the spatial pattern of the graph. This problem can be alleviated by adjusting the number of significant connections to be identical for both groups as suggested by Stam et al. [29]. The changes in *C* and *L* as a function of average number of edges connected with each node, *K*, were observed, and the optimal value of *K* was determined so that the difference between two groups was maximized.

In order to observe whether the GBPS pattern corresponds to the ‘small-world network’ [30] which is known to be the optimal structure for interregional communication [31], [32], we applied a method proposed by Sporns and Zwi [33], which is based on the fact that the small-world network has considerably higher *C* and shorter *L* compared to random network, and lower *C* and longer *L* compared to regular network [30]. 50 regular and random networks with identical degree distribution as the experimental GBPS network were generated for each subject [33]. The *C* and *L* of the experimental network were compared to those of the generated regular and random networks. Association between the GBPS network characteristics and the clinical measures was observed by Pearson's correlation coefficients between the graph-theoretic measures and clinical measures within each group.

## Results

### Demographic data

Demographic and sleep-related variables are presented in Table 1. Average age and years of full-time education were matched between RLS and control groups. The mean IRLS score of patients was 21.1±7.4 (range; 9–35). Thirteen patients (76.4%) complained poor sleep quality and nine (56.2%) complained insomnia. Mean serum ferritin level was 43.6±41.0 µg/ml (range; 1.5–164.2). Four patients showed family history of RLS in the first degree of relatives. SSS scores were not significantly different between two groups both after (*t*-test, *t* = 1.631, *p* = 0.126) and before (*t*-test, *t* = 1.535, *p* = 0.148) the experiment. However, PSQI, ESS and ISI scores were significantly different between two groups (*t*-test, *t* = 3.706, *p* = 0.001 for PSQI, *t* = 2.423, *p* = 0.022 for ESS, *t* = 3.319, *p* = 0.004 for ISI).

**Table 1:** Demographic data and sleep-related variables.

	RLS	Control	*p*
N	17	13	
Age (years)	53.7±9.6	54.6±7.6	NS
Education (years)	12.1±3.3	9.8±3.2	NS
FHx of RLS (%)	23.5	-	-
Ferritin (µg/ml)	43.6±41.0	-	-
IRLS	21.1±7.4	-	-
SSS before	2.2±1.0	3.0±1.2	NS
SSS after	2.1±1.1	3.2±1.6	NS
PSQI	7.9±3.2	4.4±1.4	0.013
ESS	6.8±4.4	3.6±1.8	0.005
ISI	10.9±8.2	3.5±2.5	0.002

Abbreviations used: RLS, restless legs syndrome; IRLS, International RLS severity scale; FHx, family history; SSS, Standford sleepiness scale; PSQI, Pittsburgh Sleep Quality Index; ESS, Epworth Sleepiness Scale; ISI, Insomnia Severity Index; NS, not significant.

### Behavioral Response

Response time (RT) was significantly slower for the patients compared to controls (controls: 382.7±32.6 ms, RLS patients: 425.4±40.3 ms, *t*-test, *t* = −3.116, *p* = 0.004). Response accuracy was 98.2% in patients and 99.1% in controls, which was not significantly different. The bothersomeness during visual oddball task was much greater for the RLS patients than controls (6.1±3.2 vs. 0.2±0.6, *t*-test, *t* = −5.147, *p*<0.001).

### Gamma-band activity

**Figure 1:**
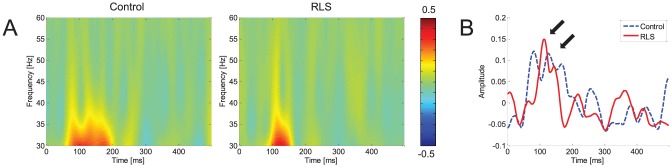
Evoked gamma-band activity. (A) Time-frequency activation patterns of evoked (phase-locked) GBA. (B) The time course of evoked GBA (in 30–50 Hz bands). These were obtained by averaging the time-frequency maps from 9 electrode sites. At the 80–200 ms epoch, remarkable increases in evoked GBA were observed for both groups. There was no significant difference in the amplitude of evoked GBA between groups, and locations. The latency of evoked GBA was also not significantly different between groups, and locations.

Figure 1*A* shows the time-frequency activation pattern of averaged event-related potential (i.e., ERSP of evoked activity), which was obtained by averaging the time-frequency maps for 9 electrodes (F3, Fz, F4, C3, Cz, C4, P3, Pz, and P4). The time-course of evoked GBA (30–50 Hz) is presented in Figure 1*B*. The grand-averaged amplitude and latency of GBA at ROIs for each group are presented in Table 2. Prominent increases in evoked GBA were observed during 80–200 ms for both groups. However, the evoked GBA amplitude was not significantly different between normal and RLS groups (*F*(1,28) = 0.031, *p* = 0.86), and locations (*F*(2,28) = 1.557, *p* = 0.217). The latency of evoked GBA was also not significantly different between groups (*F*(1,28) = 1.422, *p* = 0.236), and locations (*F*(2,28) = 0.770, *p* = 0.466).

**Table 2:** Grand-averaged amplitude and latency of GBA.

	Evoked GBA				Induced GBA			
	Amplitude (a.u.)		Latency (ms)		Amplitude (a.u.)		Latency (ms)	
ROI	Control	RLS	Control	RLS	Control	RLS	Control	RLS
Frontal	0.07 (0.19)	0.04 (0.15)	131.54 (30.20)	134.02 (18.98)	0.83 (0.79)	−0.42 (0.85)	302.82 (25.72)	346.67 (7.03)
Central	0.10 (0.22)	0.08 (0.17)	132.0 (16.18)	125.93 (21.61)	0.98 (0.67)	−1.68 (0.96)	289.94 (11.45)	352.79 (2.81)
Parietal	0.13 (0.15)	0.14 (0.18)	142.95 (22.95)	129.46 (23.15)	0.80 (0.65)	−1.12 (0.62)	310.90 (19.79)	342.30 (10.11)

All values are mean and standard deviation (in parenthesis) of GBA.

Abbreviations used: GBA, gamma-band activity; a.u., arbitrary unit; RLS, restless leg syndrome; ROI, region of interest.

**Figure 2:**
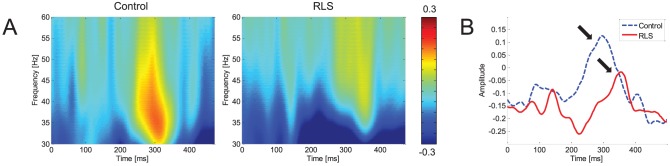
Induced gamma-band activity. (A) Time-frequency activation patterns of induced GBA. (B) Time courses of averaged induced GBA (in 30–50 Hz bands). These were obtained by averaging time-frequency maps from 9 electrodes. At the 250–400 ms epoch, prominent increases in induced GBA were observed for both groups. However, the induced GBA amplitude was significantly different between groups, and locations. Its latency was also significantly different between groups, but not between locations. *Post-hoc* pairwise comparisons revealed that the significant reduction of induced GBA was observed in RLS patients compared to normal controls. The latency of induced GBA was significantly delayed in RLS group.

In contrast to the evoked GBA, the induced GBA showed significant alteration in RLS patients. Figure 2*A* and *2B* show the time-frequency activation pattern and time course of the non-phased locked, induced GBA. Considerable increases were observed at 250–400 ms, when the induced GBA was peaked at lateral frontal and posterior regions for both groups. The induced GBA was significantly weaker for RLS group. The amplitude of induced GBA was significantly different between groups (*F*(1,28) = 139.781, *p*<0.001), and locations (*F*(2,28) = 3.973, *p* = 0.022). Its latency was also significantly different between groups (*F*(1,28) = 139.781, *p*<0.001), but not between locations (*F*(2,28) = 1.200, *p* = 0.306). In addition, the groups×location interactions were significant for both amplitude and latency of the induced GBA (amplitude: *F*(2,28) = 6.162, *p* = 0.003; latency: *F*(2,28) = 10.685, *p*<0.001).

*Post-hoc* pairwise comparisons revealed that the induced GBA amplitude was significantly reduced in RLS patients compared to normal controls in frontal, central, and parietal sites (*p*<0.001 for all three regions). The latency of induced GBA was significantly delayed in RLS group in all three regions (*p*<0.001 for three regions). The most significant inter-group difference in induced GBA amplitude and latency was found in central region (mean difference of induced GBA amplitude: 2.66 at central, 1.93 at parietal, and 1.25 at frontal region; mean difference of induced GBA latency: 62.86 ms at central, 43.85 ms at frontal, and 31.41 ms at parietal region).

All the clinical measures were not significantly correlated with the GBA amplitude for both RLS patients and controls.

### Gamma-band phase synchronization

**Figure 3:**
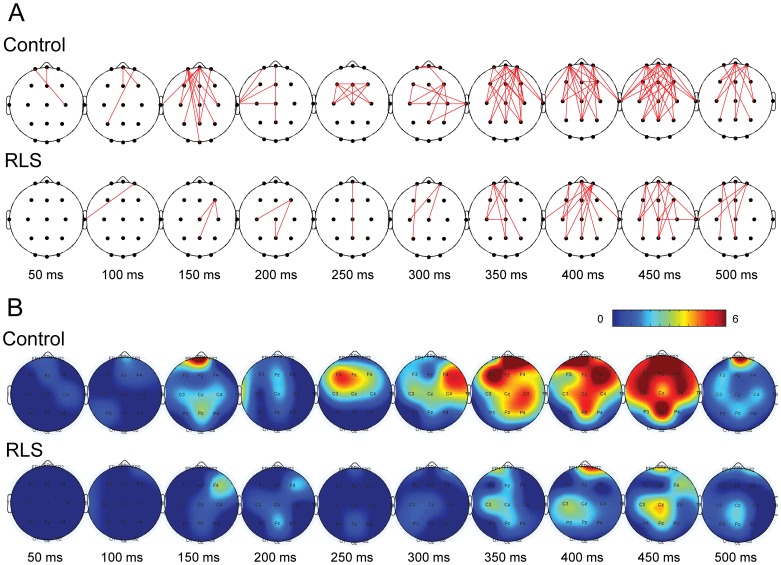
Spatiotemporal pattern of gamma-band phase synchronization (GBPS). (A) Temporal evolution of GBPS pattern. Upper and lower panels correspond to control and RLS groups, respectively. The time (ms) indicates the midpoint of the 50 ms interval from which the phase synchronization pattern was obtained. Significant GBPS was observed mainly between the anterior and posterior regions along the midline. However, the GBPS was overall weaker in the RLS group throughout the task performance. The largest difference between two groups was observed at 300–500 ms epoch. At this epoch, although the GBPS remained elevated for both groups, the numbers of significant pairs was much smaller in RLS group compared to normal group. (B) Topographical maps of the number of significant connections at each electrode derived from GBPS maps. Considerable inter-group differences were observed at 300–500 ms epoch. The numbers of significant GBPSs at each electrode was apparently reduced in RLS group compared to normal group at this epoch, especially at the frontal region.

Figure 3*A* shows temporal evolution of the spatial pattern of GBPS. The upper and lower panels represent the control and RLS groups, respectively. The solid lines denote significant increases in GBPS determined by the two criteria described above. Significant GBPS was observed primarily between the anterior and posterior regions along the midline. Overall, the GBPS was weaker in the RLS patients throughout the task performance (Table 3). The inter-group difference was highest at 300–500 ms epoch when fewer electrode pairs showed significant GBPS in the RLS group. Starting at ∼300 ms, the GBPS remained elevated for both groups. However, the number of significant pairs was much smaller in RLS patients than in normal controls. Figure 3*B* shows the topographical maps of the number of significant connections at each electrode derived from GBPS maps shown in Figure 3*A*. Considerable inter-group differences were observed at 300–500 ms. The number of significant connections to each electrode was apparently reduced in RLS patients compared to normal controls during this period, especially at the frontal region.

**Table 3:** The number of significant connections of GBPS pattern.

Time (ms)	50	100	150	200	250	300	350	400	450	500
Control	2	3	16	6	10	15	30	30	38	13
RLS	0	1	3	3	1	3	8	14	14	7

**Figure 4:**
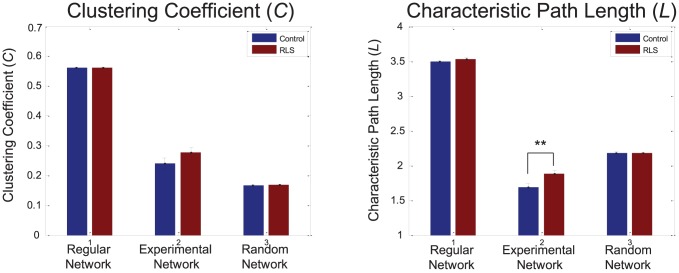
Graph theoretical analysis of gamma-band phase synchronization (GBPS) pattern. Left and right panels show clustering coefficient (*C*) and characteristic path length (*L*), respectively. These graph theoretical indices were obtained by adjusting the number of significant connections to be identical for both groups at 300–500 ms epoch. The optimal value of the degree *K* was determined to be 3–3.5. The experimental GBPS networks for both groups were found to be small-world networks which show *C* between the regular and random networks and *L* similar to random network. However, the spatial pattern of GBPS was closer to the small-world network for the control group compared to RLS. The *L* of the RLS group was significantly longer compared to normal group (**: *p*<0.005).

All the values are the number of significant connections of GBPS maps denoted in figure 4*A*.

Abbreviations used: GBPS, gamma-band phase synchronization; RLS, restless leg syndrome.

Inter-group differences in GBPS were more clearly revealed by graph theoretical analyses. Figure 4 shows graph theoretical measures of the GBPS map obtained by adjusting the number of significant connections to be identical for both groups at 300–500 ms. Following the guideline by Stam et al. [29], the optimal range of the degree *K* was determined to be 3–3.5. As described above, a network corresponds to a small-world network if its *C* is higher than that of the random network, its *L* is similar to that of the random network, and both its *C* and *L* are smaller than those of the regular network. Therefore, Figure 4 clearly shows that the GBPS networks for both normal controls and RLS patients correspond to small-world networks. However, the spatial pattern of GBPS was closer to the small-world network for the control group than the RLS group. In particular, the *L* of the RLS group was significantly longer than that of the control group.

The sleep-related measures were not highly correlated with the GBPS. We also found that ferritin levels were not highly correlated with GBA or GBPS in general, but the characteristic path length (*L*), which represents global connectivity of GBPS, was fairly correlated with the ferritin level (*r* = −0.43).

### P300 ERP component

**Figure 5:**
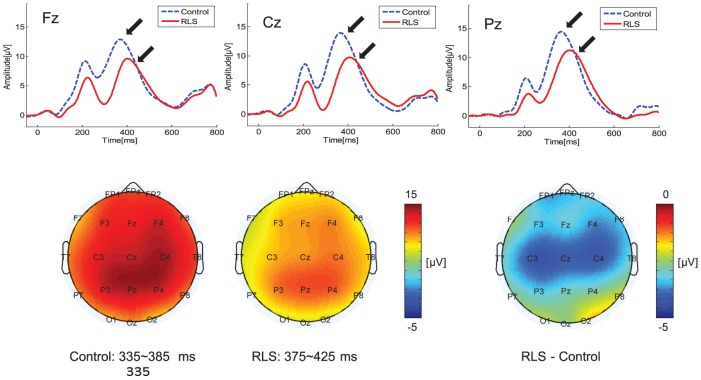
Event-related potential (ERP) waveforms and topography of the P300 component. Upper panels show grand-averaged ERP waveforms from 3 midline electrodes (Fz, Cz, and Pz), and lower panels show topographies of voltage distributions at P300 epoch. P300 was widely distributed in centroparietal regions at 350–450 ms poststimulus, as indicated by arrows in upper panels. For RLS group, the marginally significant reduction of P300 amplitude and significant delay of P300 latency were observed. *Post hoc* pairwise comparison revealed that P300 amplitude was significantly reduced for RLS group at frontal and central regions compared to normal group.

Figure 5 shows grand-averaged ERP waveforms and topographical maps of the voltage distribution at the P300 epoch. P300 was widely observed in parietocentral regions at ∼350–450 ms poststimulus, as indicated by arrows. P300 amplitude was lower in RLS patients than controls, and this difference was marginally significant (*F*(1,28) = 3.468, *p* = 0.073). P300 latency was significantly delayed in RLS patients (*F*(1,28) = 14.375, *p*<0.001). Pairwise comparisons of P300 amplitudes at different locations showed that P300 amplitudes for RLS patients were significantly decreased in frontal and central regions compared to controls (frontal: *p*<0.028, central: *p*<0.040).

## Discussion

Previous neuropsychological tests have revealed that RLS patients have cognitive dysfunction, particularly with respect to prefrontal functions, such as in the trail making test and verbal fluency [4], [5], [34]. In this study, it was shown that noticeable differences were present between normal controls and RLS patients in GBPS and induced GBA in addition to the ERP difference observed in our recent studies [25], [35]. This supports the hypothesis that abnormal cortical activity, particularly altered neural synchrony, may be underlying cognitive deficits observed in RLS patients. Although we did not employ conventional tests for cognitive function, the subjects performed an oddball task, which involves important cognitive processing such as attention and working memory. Also, the EEG-based measures of neural activity, such as the P300 ERP, gamma-band power, and interregional phase synchrony, are well-known to be closely related to cognitive function from recent cognitive neuroscience studies [12], [24], [36]–[41]. Along with the distinct degradation in behavioral scores (accuracy and response time), we judge that the changes of those EEG-based measures in RLS patients with respect to the normal controls should indicate cognitive dysfunction. We expect that our study provides a meaningful basis for further study to confirm the cognitive dysfunction in RLS and consequential changes in neural activities.

The most remarkable feature of functional connectivity was strong anterior-posterior GBPS along the midline in both groups. Simultaneous activation of anterior and posterior regions has been previously observed during oddball tasks by neuroimaging studies [42]–[44]. For example, McCarthy et al. [42] showed that midfrontal and inferior parietal regions were activated during visual oddball tasks. The anterior-posterior coupling observed in our study may reflect an association between these task relevant regions; however, it is not appropriate to pinpoint the location of neural activities synchronized in gamma-band due to the limitation in spatial resolution of EEG. Our findings are, however, in line with several previous studies which have shown that anterior-posterior interactions play an important role in information processing during task execution [45]–[47]

In the present study, diminished GBPS in RLS patients were observed throughout the task performance. An abrupt increase in GBPS was found at ∼300 ms poststimulus, i.e., the epoch of P300, in both groups, and it remained high throughout the task. However, it was much weaker in RLS patients, and the latency of GBPS peak was also delayed. The differences between normal and RLS patient groups appear to be similar for P300 and GBPS in that both were weaker and delayed in RLS patients. It is also remarkable that the changes observed in RLS patients are comparable to those associated with increased task difficulty repeatedly observed in our recent studies [25], [35].

Considering the context updating hypothesis of P300 activity [38], the neural activity in this epoch may be dedicated to updating or renewing the representation of the environment within working memory triggered by external stimuli and mediated by attention [24]. According to this hypothesis, the neural representation of the stimulus environment is updated when the incoming stimulus is incongruent to the memory contents and the subject allocates the attentional resources to the stimuli, and then P300 component is generated [39]. And the P300 was reported to be reduced and delayed when greater amounts of attentional resources were required [40], [41]. The reduced GBPS observed in RLS patients during the P300 epoch may thus reflect increased demand for attentional resources compared to control group. This was more clearly seen from the connectivity between frontal and other regions shown in Figure 3*B*. Lower and delayed induced GBA in RLS patients during P300 epoch, which is in sharp contrast with the unchanged evoked GBA during the early period dedicated to sensory processing, also suggests that attentional control of working memory update may underlie the differences in cortical information processing between the two groups [24]. Considering that the frontal region is an important part of cortical network subserving goal-directed (top-down) selection of stimuli and response [48], the pattern of reduced GBPS in RLS may reflect dysfunction in top-down selection for stimuli and responses.

Altered functional connectivity in RLS patients may be caused by structural alterations in neural networks. Significantly weaker interregional phase synchrony was observed in schizophrenia patients during a simple cognitive task [49], which was thought to be resulting from disruptions in the volume and organization of the anatomical connectivity caused by a widespread reduction in gray matter volume. Considering that several previous neuroimaging studies revealed significant alterations in both gray and white matter in RLS patients [9], [50], the alterations in GBA and GBPS observed in our study may also reflect abnormal structural alterations in cortical networks associated with RLS. However, it is expected that this structural changes may come along with altered cognitive function. It is also known that RLS is accompanied with dopaminergic dysfunction [51], which induces abnormal neural synchrony [52], [53]. Thus, the reduction in local and global neural synchrony in RLS observed in this study may be due to the dopaminergic dysfunction in RLS patients [49].

The pattern of GBPS can be regarded as a graph consisting of nodes and edges, enabling evaluation by graph theoretical analysis [27], [28], [30]. By calculating local and global connectedness measures, we found that the pattern of GBPS was closer to a ‘small-world network’ for normal subjects than for the RLS patients. The small-world network is characterized by a high degree of local connections and smaller path lengths, which is regarded as an efficient architecture for interregional communication. Thus, the graph theoretical analysis has been useful in providing quantitative data demonstrating that neural activities across cortical regions are impeded in RLS patients, which may explain the observed cognitive deficits. Neuroimaging data suggest that RLS is a neural network disorder [50], [54], [55]. However, previous studies did not explore direct functional relationship between brain regions. Furthermore, those studies focused on subcortical and sensorimotor network in RLS patients. In the present study, for the RLS patients, GBPS predominantly reduced in the connection between frontal and other regions including parietal region (see Figure 3*B*). Our previous ERP study showed decreased P300 amplitude in frontal regions in RLS patients compared to control subjects [6], which is also in accordance with previous reports suggesting frontal lobe dysfunctions in RLS patients. Thus, the result of current study showing reduced GBPS between frontal and other regions is in line with neuropsychological, neurophysiological and neuroimaging studies. It can be also conjectured that RLS has neural network disorders including both cortical and subcortical structures.

A recent neuroimaging study reported that iron insufficiency in the brain occurred with RLS [56]. Falkingham et al. (2010) showed that the iron supplementation improved attention and concentration in adolescents and women [57]. In this respect, it is notable that the characteristic path length, which represents global connectivity of GBPS, was fairly anti-correlated with the ferritin level (|*r*| = 0.43), in spite of general lack of correlation between ferritin and GBA or GBPS. This result suggests that the RLS patients with low iron status would show weak global interregional functional connectivity, which may be accompanied with cognitive deficit. This is also in line with our own finding showing significantly reduced path length in RLS patients. Further analysis with more subjects and a sleep-deprived control may help to ascertain the correlation between iron status and the interregional functional connectivity characteristic path length.

There are several potential limitations in our present study. It is not possible to dismiss the possibility of the insomnia as a factor producing cognitive deficits and changes in GBA and GBPS, since we did not use a matched sleep-deprived control. All the sleep-related clinical measures were significantly different between RLS patients and controls, implying sleep abnormality in RLS patients. Thus, the cognitive dysfunction in RLS patients in our findings may result from the sleep disruption. However, we anticipate that the lack of direct correlation between the GBA/GBPS, which are recognized as neural correlates of cognitive function, and the sleep-related measures suggests that the changes in neural activity are not directly caused by sleep deprivation. In addition, we could not find the significant difference between SSSs measured before and after the experiment, also between the patients and controls. This implies that the cognitive dysfunction in RLS patients may result from the alterations in neural synchronies by the pathophysiological mechanisms as well as the sleep deprivation. For further studies, it should be necessary for comparing the alterations in neural activities for RLS patients with those for sleep-deprived controls.

In conclusion, our results support that cognitive dysfunction in RLS patients may result from less efficient cortical information processing due to the reduced interregional neuronal synchrony as well as alterations in local activity.
